# Examining Truth Regimes Reveals How Local Communities View Flooding and River Management in the Lower Missouri River Basin, USA

**DOI:** 10.1007/s00267-025-02110-8

**Published:** 2025-01-11

**Authors:** Angela J. Catalano, Damon M. Hall, Gerardo M. Gentil

**Affiliations:** 1 Marine and Environmental Sciences, Northeastern University, Boston, MA USA; 2 School of Public Policy and Urban Affairs, Northeastern University, Boston, MA USA; 3 Coastal Sustainability Institute, Northeastern University, Nahant, MA USA

**Keywords:** Flood risk management, Community engagement, Decision-making, Water resources, Stakeholder engagement, Misinformation

## Abstract

Riverine flooding is increasing in frequency and intensity, requiring river management agencies to consider new approaches to working with communities on flood mitigation planning. Communication and information sharing between agencies and communities is complex, and mistrust and misinformation arise quickly when communities perceive that they are excluded from planning. Subsequently, riverfront community members create narratives that can be examined as truth regimes—truths created and repeated that indicate how flooding and its causes are understood, represented, and discussed within their communities—to explain why flooding occurs in their area. To better understand community perceptions of river management related to repeated flooding, we employed a qualitative methodology of semi-structured interviews with 112 community members in 3 communities on the Missouri River, USA. Discourse analysis of the interviews revealed three dominant truth regimes that shape perceptions of river management in these communities: (1) upstream reservoir releases are driven by recreational aims, such as fishing and boating within reservoirs, instead of downstream flood control; (2) endangered species protection surpasses other river values and flood management; and (3) river navigation for commerce is no longer prioritized. For environmental managers, understanding the truth regimes circulating within local affected communities can help moderate mistrust of and frustration with governing bodies, guide project messaging to disarm false truth regimes, and improve the communication of river science, management options and policy implementation.

## Introduction

Over the past 30 years, flooding has constituted 47% of weather-related disasters globally, affecting over 2 billion people (Wahlstrom and Guha-Sapir 2015; IPCC 2023). With riverine flooding events across the globe projected to increase in frequency and intensity (McDermott 2022), nations are developing new approaches to mitigation and adaptation policies to address the increasing complexities of natural hazards (Ward et al. 2020; Ulibarri et al. 2022). Creating policies to prepare for and recover from disasters like riverine flooding is challenging because of the unprecedented nature of extreme weather events and uncertainty of future flood severity (Merz et al. 2021; Kreibich et al. 2022; Klijn et al. 2022). Flood resiliency, defined as the capacity for a system to adapt to or resist flooding events that disrupt day-to-day functions of a community, has become a popular approach to river management (Batica and Gourbesville 2016; McClymont et al. 2020). River management policies require multi-level governance coordination between national and sub-national agencies and riverfront communities (Brondizio et al. 2009). Differences in infrastructure, local and regional economics, population density, and competing community priorities make policies for environmental management agencies difficult to execute and communicate at the local level (Consoer and Milman 2018; Henderson et al. 2020). Addressing these difficulties requires meaningful engagement with the communities affected by river management policies.

Involving the public in the science–policy interface often showcases divisions among scientists, policy makers, and communities; these rifts are made worse when shared values and desired outcomes are not clearly defined (Griffin et al. 2008; Colloff and Pittock 2019). As a result, people create narrative explanations for drivers of local flooding problems that allow them to reconcile and cope with this complexity (Seeger and Sellnow 2016). Conflicting interests in water resources management, such as agriculture, ecological restoration, and navigation, can destabilize policy approaches (Breen et al. 2018; Horton et al. 2019), resulting in siloing solutions based on specific interests, with less focus on the shared water issues at hand (Breen et al. 2018). Conflicting interests in shared resources management also lead to perceived vulnerabilities of the state’s ability to address climate change-related impacts, such as future floods (Miller et al. 2023). Comprehensive river management requires input from those tasked to manage it and the communities that are affected by management decisions.

Community members who perceive themselves to be excluded from decision-making conversations may attempt to reclaim power by establishing locally created truths that reflect their experiences and make sense of disasters and agency responses. These assembled truths give a sense of agency—personal control—in contexts of grave uncertainty (Afifi et al. 2012; Seeger and Sellnow 2016; Emerson et al. 2021), but they do not need to be verifiably true. The context for these truths provides insights into what communities believe is affecting them. These truths can be gathered and examined to support local flood resiliency planning and environmental management, as well as to identify misinformation within a community (Farrell et al. 2019).

In this paper, we share findings from a qualitative study that gathers and analyzes local narratives regarding river management and flooding. We apply a truth regimes analytical lens derived from social theorist Michel Foucault (Foucault 1976) that examines how people craft narratives as a form of power and resistance. Using U.S. river management policies and interviews from 3 study sites on the Missouri River as examples, we demonstrate how understanding local truth regimes can inform flood risk reduction planning and river management at scale. Truth regimes showcase complexities of applying national policies at local levels. We provide recommendations for attending to truth regimes to aid environmental managers in improving communication and community engagement and avoiding misinformation and trust degradation.

## Complexities in River Management and Community Responses to Flooding

Floods impact people in many ways. Damages to homes and personal belongings, the need to navigate complex disaster recovery policies, and the psychological toll of the cumulative experience all affect people’s overall well-being (Babcicky et al. 2021; Adger et al. 2022). This trauma is exacerbated by the ways that agencies engage impacted communities, including the timelines for engagement (Houston 2012). Interactions between agencies and communities primarily occur after a disaster, when residents are reeling from loss. Flood events can reveal un- or underinsured homes, inconsistent local compliance with national flood insurance programs, and frayed recovery (Richardson 2021; Bradt et al. 2021; Kousky and Netusil 2023), which makes interactions between residents, local recovery agencies, and national agencies fraught with anger and frustration over who is responsible for recovery and future mitigation. These complexities require comprehensive approaches to better evaluate the communication gaps between communities and the governmental agencies tasked with post-disaster recovery and mitigation planning (Birkholz et al. 2014). We provide the U.S. Army Corps of Engineers’ history and current operations within the Lower Missouri River Basin as an example of the roles of a national agency in local-level flood resiliency planning and studies.

The governing agency for water resource management in the United States is the U.S. Army Corps of Engineers (USACE), which is a joint civilian–military engineering division. The USACE was granted authorization under The Rivers and Harbors Act of 1899 to protect the navigable waterways of the United States, which includes oversight of any alterations made in and along rivers. The USACE is the lead agency authorized to oversee regulations and permitting, so any proposed project in or interfering with waterways needs to be coordinated with and approved by the USACE. With the passage of environmental regulations such as The National Environmental Policy Act (NEPA) of 1970 (P.L. 91-190), The Clean Water Act of 1972 (P.L. 92-500), and The Endangered Species Act (ESA) of 1973 (P.L. 93-205), public engagement in the form of public meetings and public comment periods were deployed to better inform and interact with communities facing environmental threats. These acts call for increased public engagement between policy makers, scientists, and communities and have helped increase public environmental awareness (Gibbons 1999). Public participation, in turn, has become a best practice for environmental policy-making (Nabatchi and Leighninger 2015).

Despite required public engagement for infrastructure studies, communities struggle to assert their experiences in policy spaces aimed at improving adaptation and environmental management (McEwen et al. 2017). Trust between flood-affected communities and governmental agencies can be difficult to establish and maintain (Sandman et al. 1993; Cox 2010; Engdahl and Lidskog 2014). For example, in the United States, federal policy communication from federal agencies to the public most often happens in the form of public meetings (including in-person and virtual gatherings), but these meetings can limit meaningful public participation. Here, technical expertise is privileged (Mileti 1999; Fischer 2000). Agency experts present plans, and time at the end is reserved for questions and comments from the public. The structure of these public meetings limits how the public can participate meaningfully and may exacerbate anger or mistrust (McComas 2003). Little knowledge exchange can happen within this structure, leaving community members without much active input in contributing to the process or study (Arnstein 1969; Senecah 2004).

### Truth Regimes as a Means to Understanding Local Truths

People craft narratives—stories that retell a series of events—to make sense of their experiences (Weick 1995; Van Den Broek 2001; Bruner 2004). These narratives are perceived as true for those crafting them and serve three primary functions. First, narratives contribute to placing blame. When faced with deep uncertainty following disasters, people employ heuristics to create narratives about who is to blame (Constantino and Weber 2021). Often, communities will look to scapegoats–people or organizations to blame that is often not the source of the problem–to exert their control over the uncertainty from the experience (Rothschild et al. 2012). Second, narratives serve as explanations for how and why disasters and their aftermaths occurred. Infrequent communication or unsatisfying answers from agencies lead communities to make sense of available information, along with their own experiences, to arrange known facts, events, and actions into these explanatory narratives. Third, local narratives serve identity functions by giving the local community a sense of agency in decision-making contexts and by aiding in processing of their personal experiences (Adams and Marshall 1996). With repetition and testing, salient narratives can develop into truth regimes.

Historian and social theorist Michel Foucault (1976) articulated the concept of truth regimes to identify the role of power in the construction of knowledge (Foucault 1976). Groups, communities, academic disciplines, industry, and institutions create truth regimes that operate as dominant logics in society. The adoption and reinforcement of dominant narratives is a type of power. Foucault delineates power between the experts who generate knowledge and the people who receive it. Truth regimes are reinforced, redefined, and pervasive. Crucially, alternative views or narratives—particularly from non-experts or non-members—are actively relegated as inferior. For example, in flood risk management, the dominant truths are those accepted and disseminated by technical experts working for flood management agencies who are tasked with conducting technical work within the bounded logics of current science, legal authorization, and agency priorities and budgets. Truth regimes develop their power from the agreement on facts and values and the distinction from falsities (Fischer 2000; Latour 2004).

Expanding Foucault’s definition of truth regimes, Weir (2008) incorporates cultural framing beyond scientific discourses to explore truth and non-truth via representation and presentation of the established truth and who is speaking this truth (Weir 2008). In Weir’s conceptualization of truth regimes, there can be multiple truths that are either stable or fighting for domination. This contestation highlights how truth regimes are continually redefined by subscribers and purveyors. It also broadens the concept to include how the non-scientific community creates and re-negotiates truth, sometimes in opposition to prevailing truth regimes established by experts. For example, in post-flood recovery, local communities will use social media platforms as a decentralized place to connect or reconnect to their community, share stories, and exchange information about the event itself, recovery efforts, technical information, among other functions (Houston et al. 2015). Online platforms have created readily accessible spaces for communities to create and disseminate truth regimes.

We use Foucault and Weir’s concepts of truth regimes to analyze how communities establish local narratives to explain the causes of flooding, make sense of the USACE planning process and timelines, and interact with the technocratic approach in USACE’s river management policies. We focus our analysis on community truth-making to examine how communities and agency technocrats negotiate power. For environmental management scholarship and practice, communities’ truth regimes on river management policies offer an empirical means for analyzing power struggles between agencies and local communities. By identifying local truth regimes, agencies and communities can develop an understanding of how blame, community-crafted narratives, and community self-affirmations play a role in community–agency dynamics during flood mitigation planning processes. This understanding can lead to strategies for improving communication and collaboration for more efficient flood resiliency planning.

### The USACE and River Management in the Missouri River Basin

The Missouri River is the longest river in the United States. It is highly altered by channelization, reservoirs, and levees, which presents numerous challenges in both management and policy priorities, as well as in determining options for flood mitigation (Ferrell 1996). The USACE manages the river for its water supply, transportation, and national security values. With the interest in national electrification via hydropower and the protection of development from annual flooding, the USACE was authorized under the Flood Control Act of 1944 (P.L. 78-534) with constructing, modifying, and maintaining a system of dams and levees along the Missouri River as part of the Pick-Sloan Missouri River Basin Program (hereafter Pick-Sloan). Following the passage of the Flood Control Act, federal and non-federal levee construction along the Missouri River took place from the 1950s through the 1980s, resulting in a patchwork system of federally and privately owned and maintained levees (Hall and Catalano 2023; Catalano et al. 2024; Hall et al. 2024). Additional alterations, including wing dikes to channelize the river and dams that control river flows, have changed the geomorphology and flow regimes of the Missouri River (Jacobson et al. 2015).

One impact from these alterations is the listing of the Pallid Sturgeon (*Scaphirhynchus albus*) in 1990 as endangered under the ESA in the Lower Missouri River Basin, downstream from Yankton, South Dakota, to the confluence with the Mississippi River near St. Louis, Missouri. Biological opinions from the U.S. Fish and Wildlife Service (U.S. Fish and Wildlife Service 2000, 2003) noted that dam operations by the USACE were affecting spawning grounds and migration patterns for the Pallid Sturgeon. Despite ongoing efforts to stabilize and increase their populations (Jacobson et al. 2016; Sansom et al. 2023), the Pallid Sturgeon remains on the ESA list as of 2024.

In 2019, the Lower Missouri River Basin experienced devastating floods that altered the way the USACE engaged with riverfront communities. Flooding began in March 2019 following record snowpack, rapidly rising temperatures, and extreme precipitation (NOAA 2019). Damages topped $1 billion, and over 14 million people across five midwestern states were affected by the flood (Smith 2020). The WRDA of 2020 authorized the Lower Missouri River Basin Flood Risk and Resiliency Study (hereafter LoMo Study) to be conducted by the USACE Kansas City District and completed by 2027. Given the extent of river miles that include levee constriction along the Missouri River, studies recommended location-specific data to create comprehensive flood mitigation plans to incorporate ecological restoration and ecosystem services (Jacobson et al. 2015, 2022). This study included updated system-wide river stage and flood frequency analyses, as well as site-specific spin-off studies or problem solving for repetitive-loss communities in Jefferson City, Brunswick, and Holt County, Missouri, to be completed concurrently as part of the LoMo Study (WRDA 2020).

### How Truth Regimes Influence Flood Resilience Planning Policy

In the United States, there are two routes for how local truth regimes can influence flood resilience planning policy: the determination of the locally preferred option and the changing weight given to other social effects in large civil works development projects (U.S. Army Corps of Engineers 2013).

First, in a planning study, USACE studies several structural and non-structural options to address problems like flooding. If a community can agree on a preferred option, then that locally preferred option must be considered in USACE’s detailed technical assessment along with other structural, non-structural, and nature-based options (U.S. Army Corps of Engineers 2013). In the case of the Lower Missouri River Basin, the state of Missouri is the non-federal cost-share partner of the Missouri part of the LoMo Study and provides 50% of the funding.

Second, in conducting feasibility studies for their projects, the USACE must conduct a benefit–cost ratio study concerning national, regional, and local costs that would arise from a proposed project. Recent shifts in non-monetizable social effects, like cultural costs or benefits of a project, open the door for local input to determine how flood mitigation projects would affect the local community. In designing flood resiliency study plans, agencies must determine how they want to approach community engagement to fulfill policy requirements that focus on the social effects of a proposed project. For the USACE, overcoming a negative public perception poses a distinctive obstacle to effective public engagement.

## Methods

To examine how truth regimes factor into community engagement in flood mitigation planning, we asked: How do local communities perceive federal river management policies? How do those perceptions shape local truth regimes about flooding?

To identify a truth regime, language is analyzed to identify dominant sociopolitical discourses (Sumares and Fidélis 2011; Langston et al. 2024). Discourse here means a system of thought made of widely circulated beliefs or logics that shape behaviors, professional practices, and institutional structures. Discourses are seen in words, images, common phrases, or other forms of communication. Discourses reveal how experiences and beliefs are framed to better understand how power and resistance take shape (Van Hulst et al. 2024). In flood resiliency planning, the discourses of peer-reviewed science and codified law direct decision-making practices.

Funded by the state of Missouri, we employed a cultural inventory (Hall et al. 2021; Gilbertz and Hall 2022) of spin-off site community leaders’ experiences and opinions about flooding and flood mitigation options via conversational and confidential interviews (Young et al. 2018). Unlike a survey, interviews allow for more authentic engagement that gives participants more time and space to find the language that best captures their experiences (Harris 2017; Grace-McCaskey et al. 2021).

**Fig. 1:**
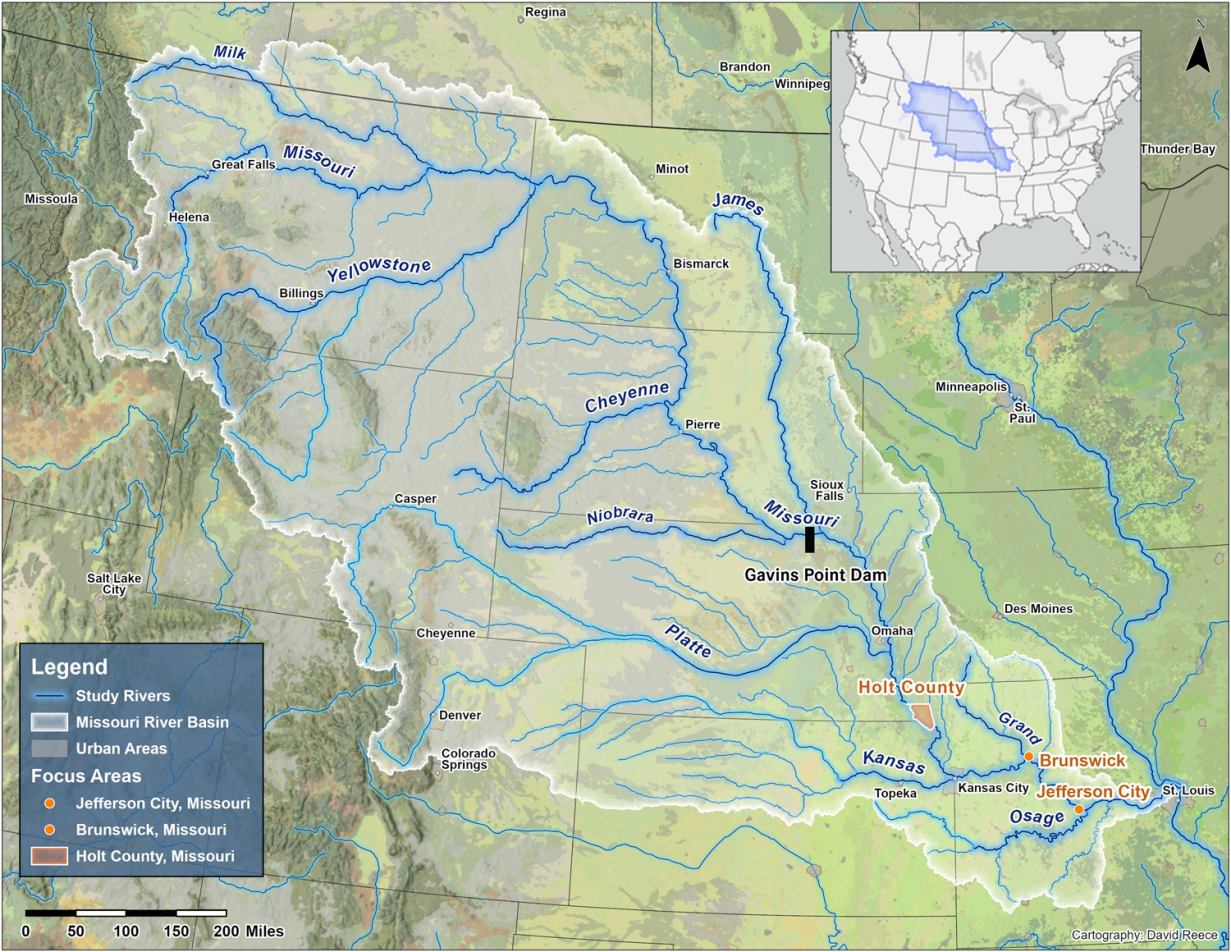
A map of the Lower Missouri River Basin, USA. Study sites are identified in orange with location names. The Gavins Point Dam, referenced in interviews, is identified in black.

We selected three sites that are part of the USACE Kansas City District’s LoMo Study: Holt County, Jefferson City, and Brunswick, Missouri (see Fig. 1 for a map of the Missouri River Basin and study site locations).

We selected these sites to assess how each community is engaged with and responding to a federal feasibility study that could result in significant changes to flood infrastructure and river management. Each site is located below the six dams on the mainstem of the Missouri River. Dam operations are managed by the USACE in compliance with the Water Control Master Manual (hereafter Master Manual) to maintain and operate water resources management for 8 purposes: hydropower, flood control, navigation, irrigation, water supply, water quality, recreation, and fish and wildlife (USACE 2018). Reservoir releases are determined by an intricate calculation that balances these 8 purposes.

Additionally, each site maintains multiple levees that have varying degrees of oversight by the USACE. All three study sites have non-federal levees that are locally sponsored and maintained but are eligible to receive levee rebuilding assistance (up to 80% of the total cost of rebuilding the levee to its pre-flooding condition) from the USACE following flood events. Holt County and Brunswick each have one federally managed and locally maintained levee in their river reach. All three sites have private levees that have no federal oversight and receive no financial or engineering support from the USACE following flood events.

Our three-person research team conducted and recorded semi-structured interviews with 112 participants (*n* = 112) across the three study sites from December 2021 through March 2024. In certain instances, interviewees wanted to have their spouse or family member, business partner, or levee district member to be interviewed at the same time. On one occasion, the group size exceeded the minimum persons for a focus group (Krueger and Casey 2015); the host invited four others. In these group interview settings, focus group dynamics occurred. In total, we interviewed 89 participants individually, and 23 participants were in small group interviews (*n* = 112). See Catalano et al. (2024) for interview protocol.

Participants were community members directly affected by flooding or those with a record of participating in flood management planning, such as local government officials and staff, levee and drainage board members, landowners and farmers located in the floodplain or floodways, business owners and industry staff whose business operations are within the floodplain or floodway, and industry members who service businesses within the floodplain or floodway. To sort based on how they were primarily affected by flooding, we categorized participants as follows: Agriculture (*n* = 35), Government (*n* = 33), Industry (*n* = 33), and Resident (*n* = 11). We recruited participants via purposive then snowball sampling (Creswell and Poth 2016). We cross-referenced contacts with the USACE public meeting attendance lists to expand our recruitment. To refine our focus on the community members’ truth regimes, we did not interview USACE staff. The interview protocol contained broad questions to assess experiences and opinions about flooding and desired flood mitigation strategies and did not explicitly ask questions that explored truth regimes.

We used our research questions, the interview protocol, and truth regimes as articulated by Foucault (1976) and Weir (2008) to develop a deductive codebook to code and analyze the transcriptions of the 112 interviewees. We developed 11 parent codes to analyze how community members expressed their engagement in the USACE’s feasibility study, as well as their opinions about what causes flooding in their area and what solutions they would like to see enacted in river management (see Supplementary Information for codebook). Our team coded every interview individually using Lumivero’s NVivo Qualitative Analysis 14.0 software and conducted a discourse analysis of the transcribed interviews (Leech and Onwuegbuzie 2011). We individually coded three test transcripts to test our codebook and compare our analyses for agreement. We listened to 5198 min of interview audio and read 2547 pages of transcribed interviews for coding. We used the NVivo software to organize, code, and analyze these data; no artificial intelligence was used in any part of this study or analysis.

Our three-person team analyzed and coded 89 interview transcripts. One of the parent codes—causes of flooding—captured what interviewees stated to be causes of flooding in their community, which is where truth regimes were expressed. After we completed the initial coding, we then analyzed the causes-of-flooding code to determine which truth regimes emerged that specifically highlighted policy decisions and implementation. We created three child codes—reservoir releases, ESA priorities, and river navigation—that emerged from the parent causes-of-flooding code to further investigate how respondents discussed how river management policies affect their communities. The first author conducted the final coding of the three child codes.

## Findings

**Fig. 2:**
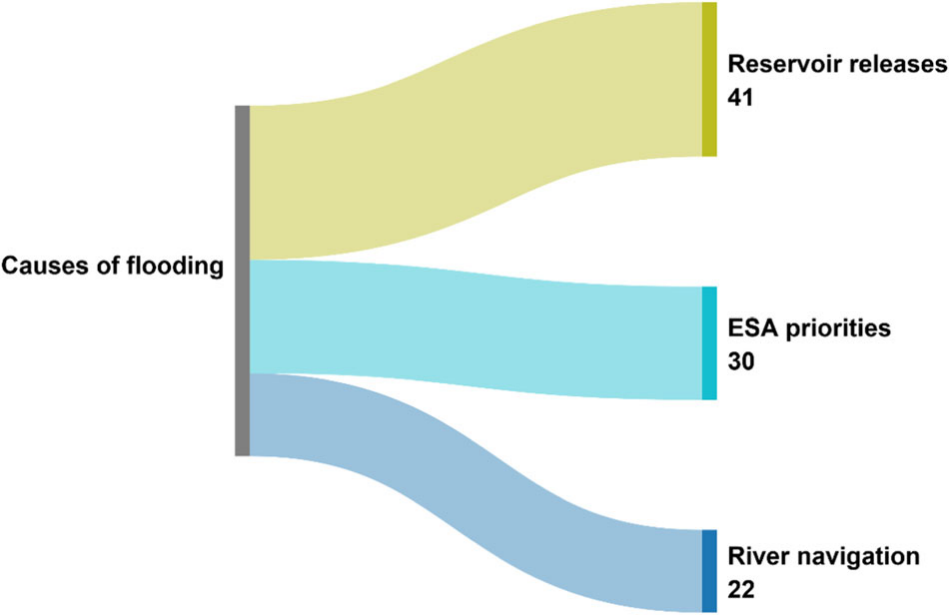
Sankey diagram of the coding flow from parent to child codes with counts. Interviewees in a transcript often referenced more than one truth regime

Eighty-seven % of the interview transcripts (*n* = 77) referenced the causes-of-flooding parent code. Our analysis found three local truth regimes—co-created explanations that perpetuate dominant narratives and diminish opposing viewpoints—all of which were related to river management and were stated by interviewees as contributors to local flooding in their communities (see Fig. 2).

We categorized the three truth regimes into these thematic headings: reservoir releases prioritize recreation over flood control, “They just manage the river for the freaking fish,” and river navigation is no longer prioritized. Each section below focuses on an individual truth regime, but we note that respondents frequently referenced more than one truth regime in a single interview. Anonymous direct quotes with category designation and abbreviated study site identifiers (Brunswick shortened to BR, Holt County to HC, and Jefferson City to JC) illustrate our findings.

### Reservoir Releases Prioritize Recreation Over Flood Control

The most prominent truth regime that locals used to explain flood severity concerned the USACE’s decision-making involved in upstream reservoir releases. Forty-six % of the coded interviews (*n* = 41) referenced the USACE reservoir releases as a source of contention within their community. The resounding narrative was that the USACE maintains higher water levels at the Gavins Point Dam to facilitate high-quality boating and fishing in the upstream reservoirs at the cost of increasing flood risk for riverfront communities downstream. This narrative is not unique to the Missouri River. Devastating floods in 2011 brought forth lawsuits in Queensland, Australia, after Wivenhoe Dam operators were found at fault for not adhering to their Operation Manual (Maslen and Hayes 2014; Chand and Akhter 2024).

Interviewees reported being aware that the USACE is required to manage the river as outlined by the Master Manual, but they did not understand how the USACE made decisions. Many participants mentioned that the Master Manual is supposed to prioritize flood control in dam system management. In the face of the unknown decision calculus of this complicated document, respondents circulated truth regimes like “the only reason they’re supposed to have them lakes [near dams] was for flood control. It wasn’t supposed to be for recreation. The recreation people, they don’t want to have a boat dock 10 foot out of the water” (Agriculture BR).

Most interviewees shared feelings of anger and resentment that their communities face more damaging floods because of the gamble of the USACE holding back more water in the reservoirs. One interviewee shared, “So what if you have to stay home and roast a hot dog instead of going fishing one day? Look at everything that got ruined just so they keep the water level the way they wanted it up north” (Industry JC).

Participants reported that they check the USACE’s Gavins Point Dam reservoir release and levels website frequently, with many stating that they check the website daily in spring and summer. One respondent stated, “I know all the levels. After the ’93 flood, I started living and breathing it. It’s just something I do all the time now” (Resident BR). The timing of reservoir releases forces residents to watch the website diligently. A participant in Holt County, the geographically closest site to the nearest reservoir, shared, “As they [USACE] regulated the Gavins Point Dam, we would be going, ‘Okay, what is the release? Today? This week?’ And all of [a] sudden, we’ve been out of the flood rains for weeks and it starts flooding again” (Agriculture HC). The volatility of water levels is a source of confusion for Lower Missouri River Basin residents. These responses indicate community members do engage with federal agency data but may not have transparency on how those data are used in decision-making contexts.

### “They Just Manage the River for the Freaking Fish”

The second dominant narrative explaining the causes of flooding was that ESA priorities are considered more important than protecting riverfront communities from impending riverine flooding (34% of the interviews, *n* = 30). Many participants shared statements like “The people concerned about the Pallid Sturgeon … those kind of people are more concerned about wildlife than they are human life” (Government HC). Many interviewees shared an “us versus them” sentiment about the Pallid Sturgeon. As one participant put it, “You won’t find any bigger conservation person than a farmer, but when they take away your livelihood, and that kind of turns your stomach a little bit” (Agriculture HC). Another participant stated that “The dinosaurs are gone, that fish needs to be gone. It’s got no value for humans, does it? You can’t say. I’ve got no problem with wildlife and stuff, but that’s ridiculous” (Agriculture BR). These truth regimes place anger against the U.S. government’s recent (1970s) river management policy changes, which are viewed as prioritizing the welfare of wildlife over that of humans.

Many participants stated they did not know how Pallid Sturgeon conservation ranked against flood control in the Master Manual, echoing their sentiments about flood releases. Noting that they experience more frequent extreme rainfall events, one participant stated,

“Uncle Sam can’t control the weather, but they can control what they do with the water after the weather. That’s where they make everybody mad because it’s not about people, it’s about fish and birds when it comes to managing the Missouri River.” (Industry BR)

Some of the respondents previously served on the MRRIC and shared feelings of disillusionment with the committee process and the focus on experts over people that live on the river. One participant reported that the USACE has new members and that:

“They’re trying to go ahead and restore the Missouri River to make it more of a wide, shallow thing. The people that are in there now are the ones that haven’t really had the experience. They’re book smart and they don’t really know how the scenarios change.” (Agriculture HC)

**Fig. 3:**
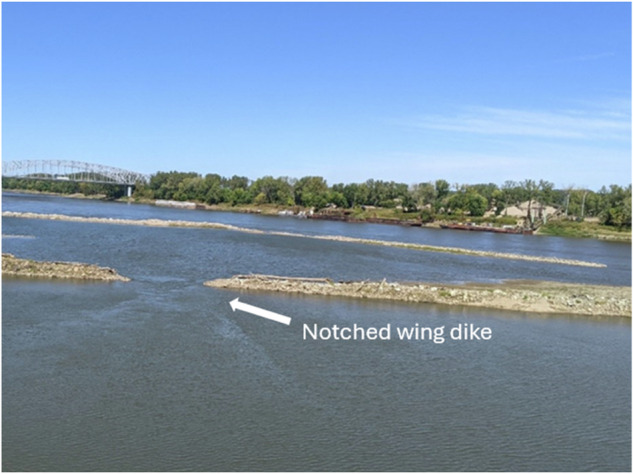
A photo of a notched wing dike in Jefferson City, Missouri USA

Respondents reported varying levels of understanding how experiments to increase Pallid Sturgeon spawning were implemented. Most stated that they were aware that the Pallid Sturgeon was still listed as endangered and were frustrated by the continuing efforts to increase the population. These experiments, most notably notching–cutting a portion from the center–wing dikes to encourage spawning, were noted by participants as failures that contributed to bank erosion and increased flooding. However, recent studies indicate that holding water in upstream reservoirs may impede Pallid Sturgeon spawning (Jacobson et al. 2016; Erwin et al. 2018) and that techniques such as notched wing dikes (see Fig. 3) may encourage Pallid Sturgeon populations when river levels are lower (Sansom et al. 2023). Much like the sentiment expressed by community members, more frequent reservoir releases may also be preferred by conservation managers to manage uncertainty in Pallid Sturgeon spawning.

One participant lamented the experimental process, stating that “bank stabilization is a huge part of flood control. And we had it at one time, and they turned and went down the other road. It didn’t fix the Pallid Sturgeon problem, but they caused a whole bunch of problems” (Agriculture JC). Many participants reported that they would prefer wildlife conservation efforts to end if that would decrease erosion and flooding.

### River Navigation is No Longer Prioritized

Building upon the two truths above, many participants noted that managing the river for navigation, as outlined in the Master Manual, no longer appears to be as important in river management as it was prior to the ESA listing of the Pallid Sturgeon. Twenty-five % of the interviews (*n* = 22) referenced bank stabilization and river navigation as causes of flooding. One participant reflected on the change in management:

“It’s so disheartening what the Corps has done. They built the river system to self-scour, to move that current to the center of the stream with the wing dikes. Then they notched them in the ‘90 s to create the Pallid Sturgeon habitat.” (Agriculture JC)

Other interviewees expressed similar frustrations with what they perceive to be a change in how the Master Manual is interpreted and implemented in the USACE’s day-to-day operations. Linking this change in management directly to flooding, a participant reported that “several of our floods we’ve had over the years, they [USACE] could control some of this flooding by just managing the infrastructure we already have…. I think that’s so important, but we don’t worry about barge navigation” (Agriculture JC).

Some participants pointed to studies (although no specific studies were referenced in our interviews) that show Pallid Sturgeons are breeding above the Gavins Point Dam, which makes it difficult for them to understand why river navigation is deprioritized in their reaches of the river. According to one participant, “Those things can breed upstream in them lakes. They’ve already proved that. So they’ve had them breeding and they found that they do, up there. Let them do that up there. Leave our river down here alone for navigation” (Agriculture HC).

Overall, interviewees pointed out how difficult it is to find consensus concerning how to manage the river. One participant shared that “it seems like the more we try and manage rivers, the more problems we cause” (Industry BR).

All 3 truth regimes narrate stories about how the federal government has changed river management practices that overlook the health and well-being of their communities. A participant summed this sentiment up by saying, “No one gives a shit about what happens to us down here” (Industry JC).

## Discussion

What emerged from our findings aligns with existing research: trust is a vital component to managing shared resources and without it, communities will establish their own local power to make sense of their experiences (Sandman et al. 1993; Coleman and Stern 2018). Truth regimes created by communities to explain the causes for environmental management problems can create roadblocks for public engagement and buy-in around management decision-making and implementation (Emerson et al. 2021). Uncovering truth regimes is not about identifying which are better or “truer” than others. Rather, the utility of truth regimes is that they reveal misalignments between community and managerial discourses, as well as differences within and among community members themselves (Weir 2008). Understanding the social dynamics, including local social norms and cooperation, supports informed resiliency planning (Boon-Falleur et al. 2022). Once identified, truth regimes can serve as a starting point for environmental managers to determine how to improve communication among technocrats and the communities they serve (Fischer 2000).

Through interviews with community members affected by flooding, we identified three truth regimes that helped these interviewees understand the causes of flooding within their contextualized experiences: timing of reservoir releases, ESA priorities around the Pallid Surgeon, and a lack of prioritization of river navigation. In our analysis, we found that the truth regimes communicated by community members affected by flooding served three primary functions: (1) to reclaim power through locally created narratives that reflect community members’ lived experiences; (2) to make sense of disastrous events in the absence of access to information; and (3) to reinforce and build upon each of the other truth regimes, thereby strengthening each other and unifying into a larger narrative.

### Reclaiming Power Through Local Narratives

For community members engaged in the LoMo Study, truth regimes act as a reclamation of power by providing their own locally created knowledge. This local knowledge aligns known facts, events, and actors into a uniform narrative that makes sense (Weick 1995). These narratives are explained in natural language and local vernacular, which contrasts with the technical terms and explanations from those outside of the community, like technocrats and scientists (Fischer 2000). In our study, farmers whose land is on the Missouri River experience the tangible impacts of flooding, which they believe to be invaluable to understanding where flooding and subsequent damage occurs most often. When technocrats present hydrological models that do not reflect their personal experiences with flooding, farmers push back on these technical explanations and models as incomplete.

Psychological and legal ownership of a place raises the stakes for community members, particularly when proposed changes threaten to undermine the social, cultural, and economic facets of the community (Matilainen et al. 2017). Following damaging floods in 2011 in the Lower Missouri River Basin, landowners sued the USACE. Plaintiffs in *Ideker Farms, Inc. et al. v. United States* (2018) alleged that the USACE was aware of the flooding and prioritized ecological restoration over flood control (Idekar Farms, Inc., et al. 2018). In 2018, the trial court determined that the USACE was responsible for taking land without just compensation and, as such, had changed priorities outlined by the Master Manual. Appeals and civil lawsuits are ongoing as of 2024. This lawsuit has contributed to mistrust between Missouri River communities and the USACE Kansas City district.

Part of the power gained from truth regimes is in organizing different people within a shared place. Truth regimes serve membership and identity functions (Adams and Marshall 1996). Adherents to the truth regime belong to a place and community. Developing truth regimes can act as a protective measure that delineates an “us-versus-them” paradigm, pitting the community against the agencies.

### Sensemaking

Functionally, truth regimes point to information deficits within communities and a failure of administrators to understand local socio-cultural particularities needed to communicate within the social context (Mishra et al. 2010; Babcicky et al. 2021). Residents used their personal experiences, combined with available agency data, to create truth regimes as a sensemaking practice to understand why their communities flood (Weick 1995). Transparency concerning how policies may restrict or limit flood mitigation options will be appreciated by riverfront communities and landowners (Lukensmeyer et al. 2011). Managers can take inventory of existing truth regimes via social science research, identify and understand the unknowns that local truth regimes explain, then incorporate explanatory communication in public meetings with communities to remove the mystery (Rowan 1992; Hall et al. 2014).

For example, our truth regime findings demonstrate that restoring Pallid Sturgeon populations and stabilizing the riverbank are viewed as two competing interests that counteract one another. The Pallid Sturgeon recovery experiments administered by the USACE and the U.S. Geological Survey failed to provide adequate explanations of experimental design methods, and the community believes them to be degrading the riverbank and stabilization efforts despite research to the contrary (Sansom et al. 2023). The presence of this truth regime indicates the current method of communication is not working and should be re-evaluated. A recent study from Liao et al. (2023) demonstrated through game-play with different communication scenarios and strategies that more direct communication improved participants’ ability to make decisions on levee infrastructure (Liao et al. 2023). There is no singular communication method that works globally, and interdisciplinary approaches to communicating can create more effective outcomes (Balog‐Way et al. 2020). Identifying local truth regimes provides information for improving or changing communication efforts and considering what options would work best given the needs of the community.

### Reinforcing and Building Upon Other Truth Regimes

Individual truth regimes do not exist in a vacuum—they build upon one another and justify the larger narratives. Truth regimes are evidence that communities want to be involved in management decisions that affect them (Fekete et al. 2021). By using the available data to support their truth regimes, these community members demonstrate that they are willing to engage with agencies outside of scheduled public meetings, and this motivation illustrates that they can be vital partners in river management planning. Moreover, data alone do not move people, and locally created truth regimes reveal the need for context in data sharing as it relates to river management decisions.

For example, the study site communities’ truth regimes evidence and reinforce the belief that they are overlooked in river management practices and that the priorities of USACE are misaligned with those of the community. Our findings demonstrate that the three truth regimes developed and circulated regionally within the over 800 river miles of the Lower Missouri River Basin. All three truth regimes relate to one another, making each truth more powerful in its salience with other local narratives. The reinforcement and pervasiveness of these truth regimes reflect their unified use by community members, in this case, to demonstrate that they both engage with the USACE websites, tools, and public meeting information and have personal experiences that conflict with what they are being told. The standardized communication tools used by agencies fail to take into account the nuanced and unique narratives built by the truth regimes communities create, resulting in power struggles, mistrust, and ineffective public outreach by agencies (Gaillard 2023).

## Recommendations and Conclusion

We recommend three approaches agencies can develop and integrate into their planning processes to help community members feel welcome and involved in decision-making in the context of complex narratives driven by interrelated truth regimes.

First, agencies should develop clear and consistent communication of *both* science and policy implementation decision-making, which will discourage misinformation and avoid mistrust. To soften the “us-versus-them” dynamic between the three study communities and the USACE, the Missouri Department of Natural Resources reviewed our findings and created in-person office hours before and after the USACE’s public meetings. These office hours allow residents to book in-person meetings to ask questions and voice their opinions one-on-one with Missouri agency staff members. The Missouri Department of Natural Resources acts not only as a locally focused resource for communities but also liaises with the USACE to share community responses to the study. This effort demonstrates to the community that the state agency, a federal cost-share partner, has a stake in this study and in the community’s perspectives as well.

Second, communities should be given space to share their knowledge and experiences in the flood mitigation planning process. Allowing this depth of participation moves public engagement beyond simply getting a community to buy in to proposed approaches and gives them agency in the decision-making process (Hall et al. 2016; Samaddar et al. 2021). Following biological opinions from the U.S. Fish and Wildlife Service (U.S. Fish and Wildlife Service 2000, 2003), the USACE formed the Missouri River Recovery Implementation Committee (MRRIC) as authorized in Section 5018 of the 2007 Water Resources Development Act (WRDA) (P.L. 110-114). This committee convened multiple interest groups to improve the USACE’s adaptive management approach and to bring in stakeholders to share their opinions and concerns with ESA management. MRRIC included stakeholder groups, tribes, and federal and state agencies that met quarterly from 2009 through 2019 to share data and provide recommendations to the USACE in an attempt to achieve consensus about how best to manage the river for everyone. The convening and meeting process revealed the challenges of negotiating among stakeholder groups in a process that is ultimately governed by bureaucracy (Errington and Gewertz 2018).

Third, effort should be made to identify and understand local truth regimes. Directly speaking to these truth regimes and the larger narratives they support can increase the legitimacy of public participation and can be used to clarify discrepancies between agency and local knowledge. Communities want to be heard and considered in decision-making that directly affects them. Meaningful engagement—engaging directly in ways that best align with local communication preferences—between local residents and agencies encourages decentralizing power and supports more informed approaches to addressing community needs in national policies (Hassenforder et al. 2020; Tambal et al. 2024).

As more agencies hope to engage with local communities effectively, residents likely benefit from being better informed about river management policies so they are more capable to meaningfully participate, increasing the likelihood of their preferences being heard and selected (Mees et al. 2016; Raikes et al. 2023). Additionally, agencies need to know if their current outreach and communication is perceived accurately to better address misinformation, disinformation, or perceived animosity (Cox 2010; Coleman and Stern 2018). Considering scale is vital to research design for collecting data prior to developing an engagement approach (Hall et al. 2012). Since federal policies look different at local scales, site-specific policy communication can be developed by investigating which truth regimes develop and circulate in a community or region. By listening to and documenting local truth regimes, agencies can identify core community values to craft or deliver information in ways that suit the community (Lewandowsky and Oberauer 2016). Tailored communication can be valuable for increasing local education, encouraging participation, and supporting acceptance of flood mitigation strategies (Enu et al. 2024).

Using data from semi-structured interviews with riverfront community members, we analyzed how local truth regimes identify power struggles over knowledge about river management between river management agencies and the communities affected. Our findings reveal that without consistent and purposeful engagement with local communities about river management implementation, agencies risk losing trust and potentially increasing misinformation when re-evaluating flood risk reduction approaches. By identifying local truth regimes and developing communication strategies that speak directly to community-crafted narratives, environmental managers may gain trust and avoid misinformation. Our study highlights a community-centered approach to understand how community members challenge agency-driven information campaigns and craft their own truth regimes. Listening to community truth regimes and developing clear and consistent communication of both data-driven science and relevant policies can improve public participation in flood mitigation planning and enhance implementation of river management policy.

## Supplementary information

Supplementary Information

## Data Availability

The data that support the findings in this study are restricted due to confidentiality agreements and are not available publicly.
