# Supplementary material for: Examining Truth Regimes Reveals How Local Communities View Flooding and River Management in the Lower Missouri River Basin, USA
Source: Environ Manage. 2025 Jan 11;75(4):982–95. doi: 10.1007/s00267-025-02110-8 (PMC11965192; doi:10.1007/s00267-025-02110-8)
Supplement: Supplementary file 1 — Supplementary Information [file 267_2025_2110_MOESM1_ESM.docx]

# Supplemental Information: Codebook

| **Parent codes for NVivo analysis** | **Description** |
| --- | --- |
| 1. Types of flood mitigation/resiliency solutions/strategies | Any references to controlling or allowing flooding; lessen damage or catastrophic flooding; connecting L-536 with Forest City federal levee; levee setbacks; flood walls |
| 1. Communication with federal and state organizations | Positive or negative communication with USACE, FEMA, SEMA, MDNR, MODOT |
| 1. Causes of flooding | Any references to dams, reservoirs, or USACE oversight of Missouri River |
| 3a. Reservoir releases | References to Gavins Point or other dams; releases or timing of releases |
| 3b. Endangered Species Act priorities | Pallid sturgeon or any references to ESA listed species; piping plover; least tern |
| 3c. Bank stabilization & river navigation | References to bank erosion control measures, dredging, commercial navigation |
| 1. Private Levees | level of protection, funding, monitoring, and repairs. Code alternative names such as farm levee, non-federal levee, agricultural levee |
| 1. Federal Levees | level of protection, funding, monitoring, and repairs. Include alternative names such as super levee; L-246 |
| 1. Role of the railroad | Anything related to railroad, BNSF or NS |
| 1. What should the Corps study? | Any mention of USACE work or planning |
| 1. Effects of flooding on the community | Residents that move out of floodplain; abandoned or low-population towns; lost revenue or business closures |
| 1. Land & property ownership & exchange | Allowing NGOs/nonprofits/governmental organizations to buy back land; allow farmers to keep land for taxable purposes if setting levee back; eminent domain; property taxes (on combine harvesters, taxed farm equipment); FEMA buyouts |
| 1. Fatigue | Expressions of fatigue from disaster, from planning, from studies |
| 1. Connection to the river | Includes mentions of connection and disconnection on individual and community level to Missouri River and connected rivers and tributaries |
